# Reduced graphene oxide/carbon double-coated 3-D porous ZnO aggregates as high-performance Li-ion anode materials

**DOI:** 10.1186/s11671-015-0902-7

**Published:** 2015-05-01

**Authors:** Sungun Wi, Hyungsub Woo, Sangheon Lee, Joonhyeon Kang, Jaewon Kim, Subin An, Chohui Kim, Seunghoon Nam, Chunjoong Kim, Byungwoo Park

**Affiliations:** WCU Hybrid Materials Program, Department of Materials Science and Engineering, Research Institute of Advanced Materials, Seoul National University, Seoul, 151-744 South Korea; School of Materials Science and Engineering, Chungnam National University, Daejeon, 305-764 South Korea

**Keywords:** Li-ion battery, 3-D porous ZnO aggregate, Reduced graphene oxide, Double coating

## Abstract

The reduced graphene oxide (RGO)/carbon double-coated 3-D porous ZnO aggregates (RGO/C/ZnO) have been successfully synthesized as anode materials for Li-ion batteries with excellent cyclability and rate capability. The mesoporous ZnO aggregates prepared by a simple solvothermal method are sequentially modified through distinct carbon-based double coating. These novel architectures take unique advantages of mesopores acting as space to accommodate volume expansion during cycling, while the conformal carbon layer on each nanoparticle buffering volume changes, and conductive RGO sheets connect the aggregates to each other. Consequently, the RGO/C/ZnO exhibits superior electrochemical performance, including remarkably prolonged cycle life and excellent rate capability. Such improved performance of RGO/C/ZnO may be attributed to synergistic effects of both the 3-D porous nanostructures and RGO/C double coating.

**Electronic supplementary material:**

The online version of this article (doi:10.1186/s11671-015-0902-7) contains supplementary material, which is available to authorized users.

## Background

Recently, metal oxides undergoing the conversion reactions have been intensively studied as promising anode materials for lithium-ion batteries since they can overcome the capacity limitation of graphite (372 mAh/g) [1-3]. Among various metal oxides, ZnO has received attention due to some advantages, such as reasonably high theoretical capacity (978 mAh/g), environmental benignity, low cost, and availability for tailoring assorted nanostructures [4-6]. However, ZnO suffers from particle fracture and loss of electrical contact arising from the morphological changes during electrochemical reactions with Li^+^ (Equations 1 and 2) [7-9]:

1 $$ \mathrm{Z}\mathrm{n}\mathrm{O}\kern0.5em +\kern0.5em 2\mathrm{L}{\mathrm{i}}^{+}\kern0.5em +\kern0.5em 2{e}^{-}\kern0.5em \leftrightarrow \kern0.5em Z\mathrm{n}\kern0.5em +\kern0.5em \mathrm{L}{\mathrm{i}}_2\mathrm{O} $$

2 $$ \mathrm{Z}\mathrm{n}\kern0.5em +\kern0.5em \mathrm{L}{\mathrm{i}}^{+}\kern0.5em +\kern0.5em {e}^{-}\kern0.5em \leftrightarrow \kern0.5em \mathrm{L}\mathrm{iZn} $$

In order for ZnO to circumvent these limitations, various nanostructures have been suggested, most of which include nanoparticles, nanowires, nanotubes, hollow spheres, core-shell structures with carbon, porous structures, nanocomposites with reduced graphene oxide, *etc*. [10-21]. Among these candidates, 3-D porous aggregates composed of nanoparticles clearly have two outstanding advantages: pores between nanoparticles act both as free spaces to accommodate the volume variations during cycling and as short diffusion paths of Li ions into the nanoparticles [22-27]. Furthermore, conformal carbon coating onto the nanoparticles is one of the well-known techniques to effectively restrain the volume change during lithiation/delithiation [28-34]. Such a carbon coating entails the use of disordered carbon while the electronic conductivity is not significant until the carbonization temperature is higher than the temperature when carbothermal reductions of metal oxides start to occur (approximately 600°C) [35,36]. Alternatively, the flexible graphene, a *sp*^2^-hybridized two-dimensional carbon layer is one of the best effective ways to enhance the anode performance of ZnO by providing high electronic conductivity and/or circumventing mechanical stresses during the electrochemical cycling [35-44].

In this study, we have focused on improving the reversible capacity and cyclability of ZnO by 3-D porous nanostructures and sequential surface modification through distinct carbon-based coating steps. The 3-D porous structures can benefit from the mesopores acting as free spaces to accommodate volume expansion during cycling. In addition, the double coating of reduced graphene oxide (RGO) and disordered carbon on both the micrometric and nanometric dimensions of ZnO aggregates, respectively, establishes a conductive network connecting the aggregates and rigid buffer layers for volume changes of ZnO nanoparticles. As a consequence, the RGO/C/ZnO nanocomposites can exhibit not only high reversible capacity with long cycle life but also enhanced rate capability.

## Methods

The 3-D porous ZnO aggregates (ZnO) were synthesized by a solvothermal method. Typically, zinc acetate dihydrate (Zn(CH_3_COO)_2_ · 2H_2_O: Sigma-Aldrich) was added to diethylene glycol ((HOCH_2_CH_2_)_2_O: Sigma-Aldrich) and heated in an autoclave at 160°C for 6 h. The as-synthesized solution was then centrifuged and washed with ethanol, and subsequently dried at 60°C [45,46].

The carbon-coated ZnO aggregates (C/ZnO) were synthesized by impregnating the as-synthesized ZnO powders in sucrose solution (sucrose:ZnO = 3:7 by weight) followed by drying and calcining them at 550°C for 3 h under H_2_/Ar (4 vol.% H_2_) atmosphere. A modified Hummers’ method was used to synthesize graphene oxide (GO), as described elsewhere [47,48]. Prior to GO wrapping, the surface modification of ZnO (or C/ZnO) was first performed by mixing aminopropyltriethoxysilane (C_9_H_23_NO_3_Si: APTES) with ZnO in ethanol dispersion for 12 h. An aqueous graphene-oxide suspension (100 ml, 2 mg/ml) was added into the APTES-modified ZnO dispersion (500 ml, 1 mg/ml) under stirring for 20 min, followed by centrifugation [43,49-52]. Thermal reduction of GO was carried out under H_2_/Ar 4 vol.% H_2_ at 550°C for 3 h.

The crystal structure and grain size of the ZnO aggregates were characterized by X-ray diffraction (XRD, D8 Advance: Bruker). The morphology was analyzed using a field-emission scanning electron microscopy (FE-SEM, SU70: Hitachi), and the carbon content was measured using a carbon, hydrogen, nitrogen, sulfur (CHNS) analyzer (Flash EA 1112: Thermo Electron Corp.). The nitrogen adsorption and desorption isotherms were obtained at 77 K (Micromeritics ASAP 2010), and the specific surface area and the pore size distribution were calculated by the Brunauer-Emmett-Teller (BET) and the Barrett-Joyner-Halenda (BJH) methods, respectively.

For the electrochemical characterization, the active materials were tested by using coin-type half cells (2016 type) with a Li counter electrode. The composition of the electrode was set to be the same for all of the samples, which consisted of an active material, super P carbon black, and a polyvinylidene fluoride binder with a weight ratio of 3:1:1, and the geometric area of the electrode was 0.71 cm^2^. Calculation of the specific capacity of the cell is carried out based on the carbon content from CHNS analysis. The specific capacity of carbonaceous materials was assumed to have the same theoretical capacity with graphite (372 mAh/g). The minor contribution from the conductive additive (super P carbon black) was excluded. The electrolyte contained 1 M LiPF_6_ in ethylene carbonate and diethylene carbonate (1/1 vol.%) (Panax Etec). Electrochemical impedance spectra (EIS) were measured using a potentiostat (CHI 608C: CH Instrumental Inc.) after 2 cycles, and the applied voltage was 0.5 V with an AC amplitude of 5 mV in the frequency range from 1 mHz to 100 kHz.

## Results and discussion

**Figure 1:**
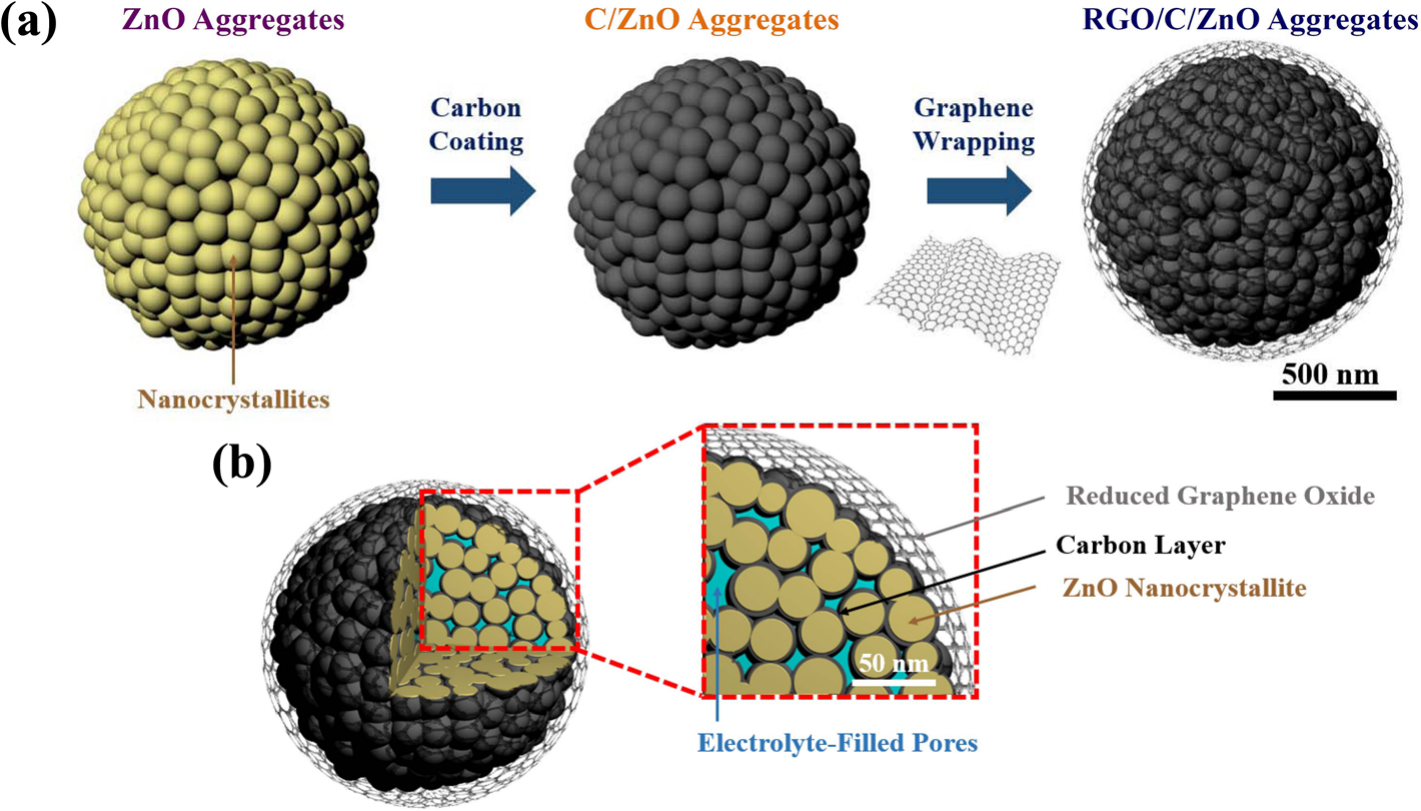
Schematic illustration for the RGO/C double-coated ZnO aggregates. **(a)** The synthesis process, via carbon coating followed by graphene oxide (GO) wrapping using electrostatic interactions, and thermal reduction of GO. **(b)** Three-dimensional view and two-dimensional cross-section view of the reduced graphene oxide/carbon double-coated ZnO aggregates (RGO/C/ZnO).

The synthetic processes for the RGO/C double-coated ZnO aggregates are illustrated in Figure 1a. The solvothermal method initially produced approximately 25-nm-sized nanoparticles which, afterwards, aggregated to the 3-D porous ZnO. After conformal carbon coating on the surface of each ZnO nanoparticle, the carbon-coated ZnO (C/ZnO) was wrapped by graphene oxide (GO) sheets. The positively charged C/ZnO prepared through the surface modification by APTES attracts negatively charged GO, thereby resulting in the GO/C/ZnO nanocomposites [36]. The final annealing process gives rise to the reduction of graphene oxide (RGO), establishing a three-dimensional network that renders well-connected electron percolation among the C/ZnO aggregates.

**Figure 2:**
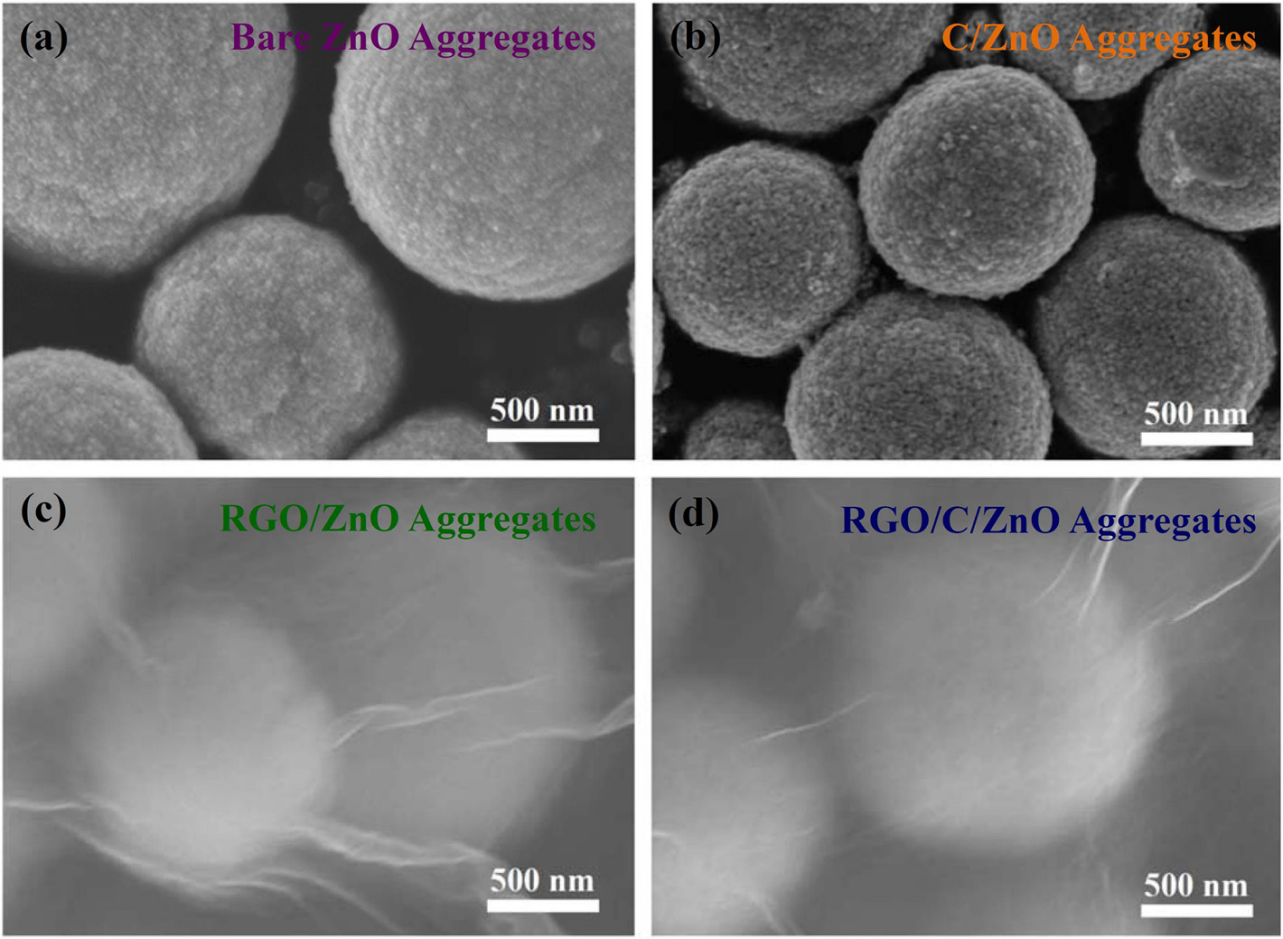
SEM images. **(a)** Bare ZnO, **(b)** C/ZnO, **(c)** RGO/ZnO, and **(d)** RGO/C/ZnO.

**Figure 3:**
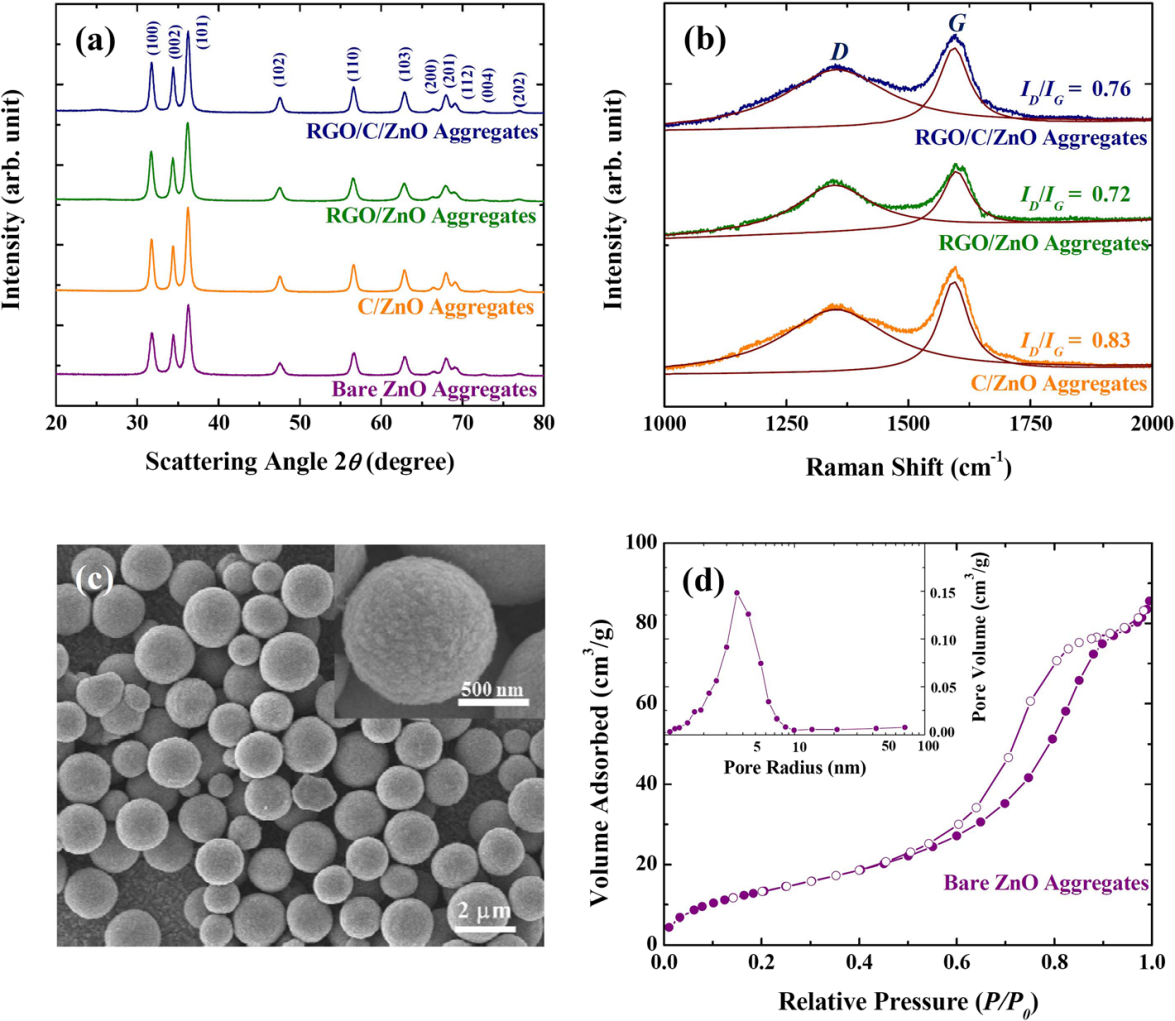
Characterization of ZnO aggregates. **(a)** XRD patterns of the RGO/C/ZnO, RGO/ZnO, C/ZnO, and bare ZnO. **(b)** Raman spectra of the RGO/C/ZnO, RGO/ZnO, and C/ZnO. **(c)** SEM images of the bare ZnO aggregates. **(d)** N_2_ adsorption/desorption isotherms of the bare ZnO aggregates. The inset shows the pore-size distribution of the bare ZnO aggregates.

The bare ZnO (Figures 2a and 3c) clearly shows porous microspheres that consist of the approximately 25-nm-sized nanoparticles. The morphology of the C/ZnO (Figure 2b) resembles that of the bare sample. Both RGO-wrapped ZnO (RGO/ZnO) and RGO/C/ZnO are covered and connected to each other by the soft RGO sheets providing facile electron conduction (Figure 2c, d) and Additional file 1: Figure S2a, b.

All of the diffraction peaks are indexed to ZnO with hexagonal wurtzite structure (JCPDS #36-1451) (Figure 3a), and the diffraction peak widths ∆*k* (full width at half maximum) were fitted using double-peak Lorentzian functions for *Kα*_1_ and *Kα*_2_. Grain sizes of the samples were estimated by the Scherrer equation [53,54] and are listed in Table 1. It can be recognized that the conformal carbon layer prevents the growth of ZnO nanoparticles during the annealing steps. The RGO sheets on the ZnO aggregates, however, were not as effective as the carbon layer, as expected, in terms of suppressing the grain growth of each nanoparticle (Table 1) [55]. The *I*(_*D*_) and *I*(_*G*_) from the Raman spectra are the ratio of defective and *sp*^2^ bonding characters of the carbon, respectively. The lower *I*(_*D*_)/*I*(_*G*_) was observed in the RGO-coated sample than C/ZnO, which indicates that RGO has the richness in *sp*^2^ bonding than the disordered carbon. This results in higher conductivity than the disordered carbon-coated samples. The Raman spectra of RGO/C/ZnO lie between C/ZnO and RGO/ZnO, proving that the RGO/C/ZnO is successfully modified by both the reduced graphene oxide and sucrose-derived carbon (Figure 3b) [56-60].

**Table 1:** **Carbon content, grain size, Raman-intensity ratio, and charge-transfer resistance of the RGO/C/ZnO, C/ZnO, and RGO/ZnO**

**Sample**	**Carbon content (wt.%)**	**Grain size (nm)**	**Raman intensity (** ***I***_***D***_ **/** ***I***_***G***_ **)**	**Charge-transfer resistance (** ***R***_***ct***_ **) (Ω cm** ^**2**^ **)**
RGO/C/ZnO	18.9	31.6 ± 7.9	0.76	60.8 ± 0.6
C/ZnO	7.6	31.3 ± 7.8	0.72	273.3 ± 1.2
RGO/ZnO	14.6	45.7 ± 17.1	0.83	99.6 ± 1.0

The porous nanostructures of the bare ZnO aggregates were also confirmed by BET and BJH (Figure 3d), showing a typical type-IV mesoporous structure [61]. The BET surface area of the ZnO aggregates amounts to 144.6 m^2^/g, and a pore distribution of approximately 3.5 nm was determined by the desorption curve (the inset of Figure 3d). The SEM image which shows a broken ZnO aggregate also indicates the porosity inside of the ZnO aggregates (Additional file 1: Figure S1), and the pores between primary particles are reflected in the BET analysis. The surface area and average pore size of the C/ZnO, RGO/ZnO, and RGO/C/ZnO are given in Additional file 1: Table S1 and Figure S3, and all the coated aggregates have mesoporous characteristics. These porous nanostructures can be beneficial both for the facile Li diffusion and free-space buffering during volume variation [22-24,58].

**Figure 4:**
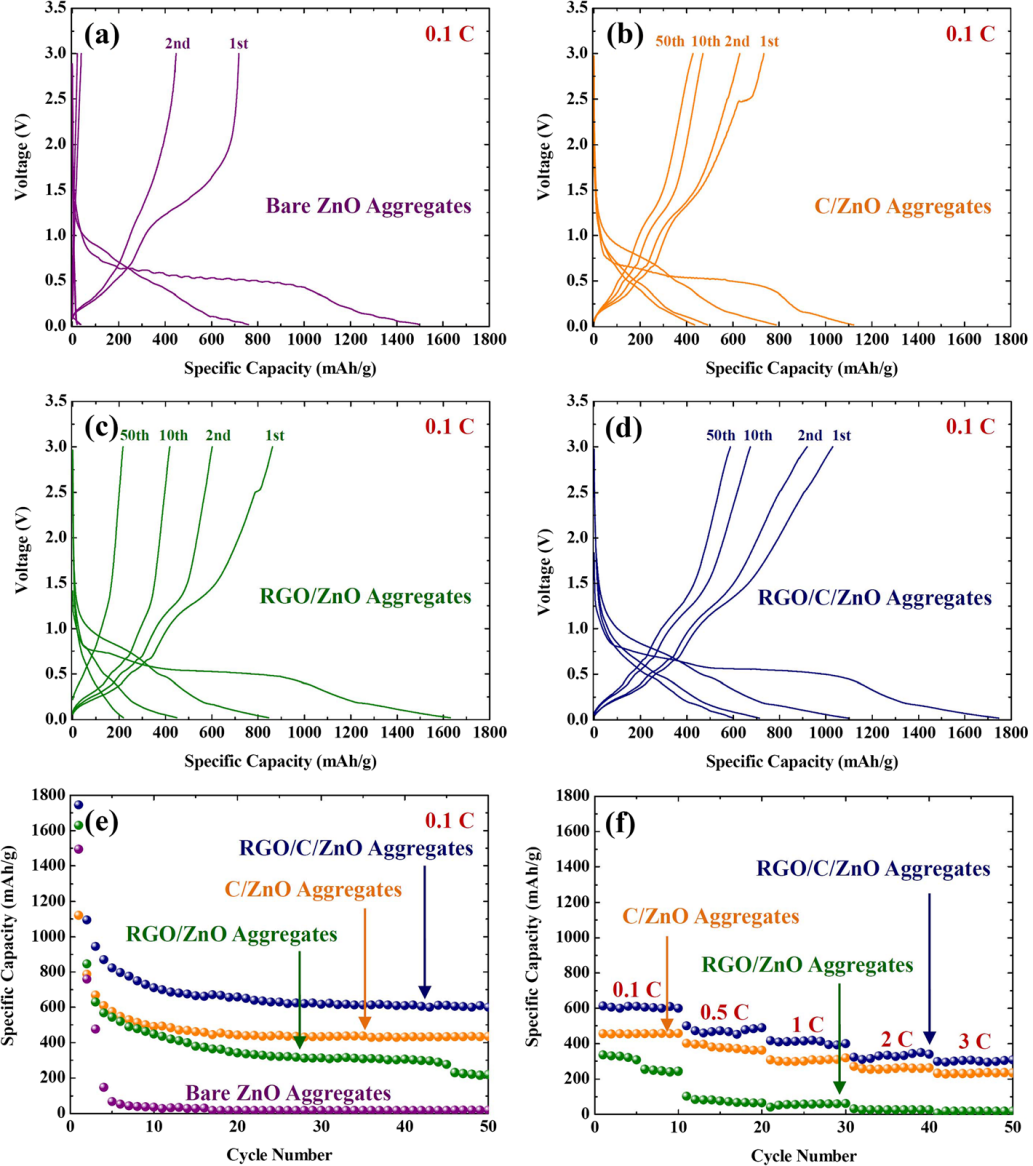
Electrochemical properties. Charge–discharge curves of the **(a)** bare ZnO, **(b)** C/ZnO, **(c)** RGO/ZnO, and **(d)** RGO/C/ZnO. **(e)** Cycle-life performances of the RGO/C/ZnO, RGO/ZnO, C/ZnO, and bare ZnO. **(f)** Rate capability of the RGO/C/ZnO, RGO/ZnO, and C/ZnO (1 C = 978 mA/g).

To identify the effects of the carbon-based modifications on the electrochemical performance, the bare ZnO, C/ZnO, RGO/ZnO, and RGO/C/ZnO were galvanostatically cycled in the range of 0.02 to 3.00 V (vs. Li^+^/Li) at a current density of 97.8 mA/g (= 0.1 C) (Figure 4a, b, c, d, e). For the first cycle, all the samples show very high discharge capacity. It is well known that side reactions with an electrolyte such as a formation of the SEI layer severely occur on the surface area of the active material under 1 V during the first discharge, which will result in the low coulombic efficiency in particular using nanosized materials [62]. Interestingly, more vigorous side reactions could be observed in the case of graphene modification [15,40]. Cyclic voltammogram (CV) curves in Additional file 1: Figure S4 confirm that only Li insertion below 0.5 V occurs with vigorous side reactions with the electrolyte. It seems that the bare ZnO suffers from a significant capacity loss only after 5 cycles. In terms of the composites, the capacity fading was more significant for the RGO/ZnO compared to the C/ZnO or RGO/C/ZnO, yielding a discharge capacity of approximately 218 mAh/g at the 50th cycle. The C/ZnO and RGO/C/ZnO, on the other hand, show more stable cycle-life performances, which indicate the carbon layers effectively inhibit the massive aggregations of Zn/ZnO nanograins during cycling. The higher reversible capacity of the RGO/C/ZnO sample (approximately 600 mAh/g after 50 cycles) than that of the C/ZnO comes from the 3-D network of graphene wrapping the C/ZnO, enhancing the electronic percolation within the secondary particles.

**Figure 5:**
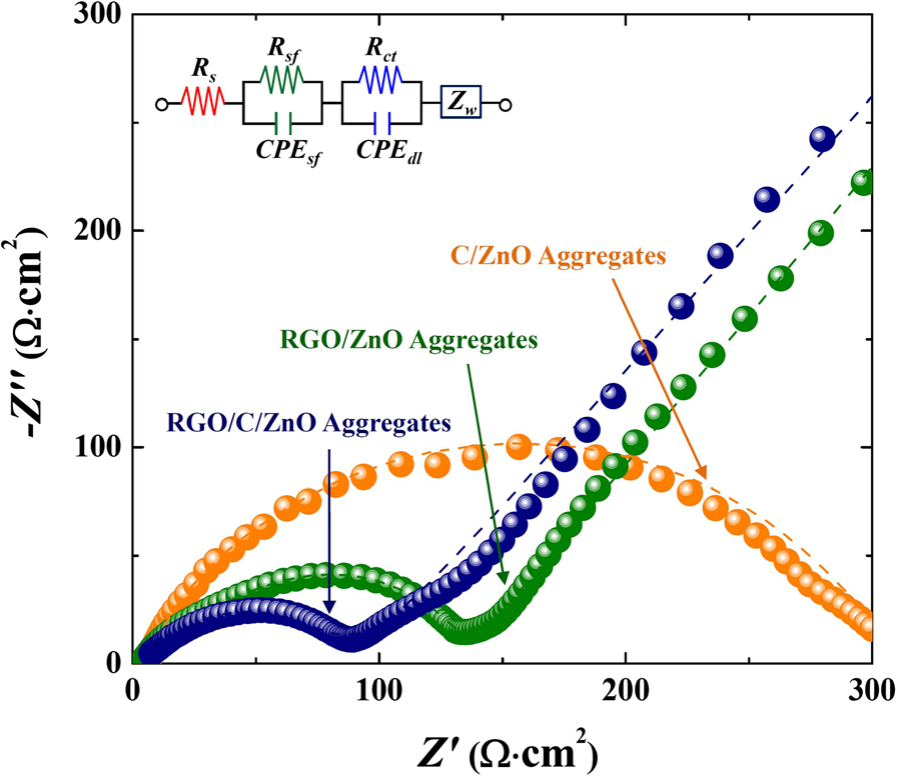
Electrochemical impedance spectra of the RGO/C/ZnO, RGO/ZnO, and C/ZnO with an applied voltage of 0.38 V after 3 cycles. The fitting lines were obtained using the equivalent circuit in the inset.

Regarding the rate capability, the RGO/ZnO shows a dramatic capacity fade with the increased current density, and the capacity is hardly observed at a current density of 1,956 mA/g (= 2 C) (Figure 4f). Meanwhile, the RGO/C double-coated ZnO and C/ZnO exhibit the revisable capacity of approximately 300 mAh/g and approximately 230 mAh/g even at a rate as high as 3 C rate (2,934 mA/g), respectively. The kinetics involved in ZnO through the modification by RGO- and/or C are evaluated by electrochemical impedance spectroscopy (EIS) with an equivalent circuit (Figure 5). The diameter of the semicircle can be approximately assigned to the charge-transfer resistance (*R*_*ct*_): the RGO/C/ZnO electrode exhibits smaller *R*_*ct*_ than C/ZnO or RGO/ZnO, indicating better electrochemical activity [57,60].

**Figure 6:**
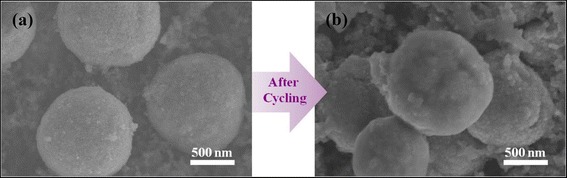
Comparison of C/ZnO electrodes before and after cycling. SEM image of the **(a)** initial C/ZnO and **(b)** C/ZnO after 10 cycles at 0.1 C rate.

The RGO/C double-coated porous ZnO aggregates exhibit good cyclability, high specific capacity, and excellent rate capability, which are attributed to both the 3-D porous nanostructures and RGO/C-double coating of aggregates. First, the approximately 3.5-nm pores can provide a space to alleviate the volume expansion during cycling. Second, the carbon coating layer on each ZnO nanoparticle can buffer the volume expansion during lithiation. Therefore, the overall morphology during cycling can be preserved without much fracture of approximately 1-μm porous aggregates, as confirmed in Figure 6. Also, the 3-D network of graphene, wrapping around the C/ZnO porous powders, enhances the electronic conduction through the aggregates.

## Conclusions

In this work, we have proposed the RGO/C double-coated ZnO nanocomposites as an anode material with excellent electrochemical properties. The 3-D porous ZnO aggregates are facilely modified through distinct carbon-based coating steps via conformal carbon coating, GO wrapping, and thermal reduction. The approximately 32-nm-sized RGO/C/ZnO nanocomposites with approximately 1-μm porous powders exhibited superior electrochemical performance, including remarkable cycle life, high reversible capacity, and excellent rate capability. The enhanced electrochemical performance arose from the combination of unique properties of the mesopores acting as free space to accommodate volume expansion during cycling, conformal carbon layer on each nanoparticle surface buffering volume changes, and conductive RGO sheets connecting the aggregates to each other. The work introduced in doubly coated ZnO can be extended to the synthesis of other novel electrodes where the cycle life and rate capability are significantly associated with their mechanical failure and appropriate electronic conduction.

## Additional file

Additional file 1:
**Supporting Information.** **Table S1.** BET surface area and average pore size of the C/ZnO, RGO/ZnO, and RGO/C/ZnO. **Figure S1.** SEM image of the bare ZnO aggregates. **Figure S2.** SEM images of the (a) RGO/ZnO aggregates and (b) RGO/C/ZnO aggregates. **Figure S3.** N_2_ adsorption/desorption isotherms of the C/ZnO, RGO/ZnO, and RGO/C/ZnO. The inset shows the pore-size distribution of these samples. **Figure S4.** Cyclic-voltammetry of (a) C/ZnO and (b) RGO/C/ZnO (0.001 to 3.0 V with the scan rate of 0.1 mV/s).

## References

[1] Poizot P, Laruelle S, Grugeon S, Dupont L, Tarascon JM (2000). Nano-sized transition-metal oxides as negative-electrode materials for lithium-ion batteries. Nature.

[2] Aricò AS, Bruce P, Scrosati B, Tarascon JM, Schalkwijk WV (2005). Nanostructured materials for advanced energy conversion and storage devices. Nat Mater.

[3] Cabana J, Monconduit L, Larcher D, Palacín MR (2010). Beyond intercalation-based Li-ion batteries: the state of the art and challenges of electrode material reacting through conversion reactions. Adv Mater.

[4] Li WY, Xu LN, Chen J (2005). Co_3_O_4_ nanomaterials in lithium-ion batteries and gas sensors. Adv Funct Mater.

[5] Belliard F, Connor PA, Irvine JTS (1999). Doped tin oxides as potential lithium ion battery negative electrodes. Ionics.

[6] Ku JH, Jung YS, Lee KT, Kim CH, Oh SM (2009). Thermoelectrochemically activated MoO_2_ powder electrode for lithium secondary batteries. J Electrochem Soc.

[7] Fu ZW, Huang F, Zhang Y, Chu Y, Qin QZ (2003). The electrochemical reaction of zinc oxide thin films with lithium. J Electrochem Soc.

[8] Kushima A, Liu XH, Zhu G, Wang ZL, Huang JY, Li J (2011). Leapfrog cracking and nanoamorphization of ZnO nanowires during in situ electrochemical lithiation. Nano Lett.

[9] Huang XH, XiaX XH, Yuan YF, Zhou F (2011). Porous ZnO nanosheets grown on copper substrates as anode for lithium ion batteries. Electrochim Acta.

[10] Shin WH, Hwang TH, Huh YS, Choi JW (2012). Electrochemically controlled nanopore and crystal structure evolution in zinc oxide nanorods. J Electrochem Soc.

[11] Park KT, Xia F, Kim SW, Kim SB, Song T, Paik U (2013). Facile synthesis of ultrathin ZnO nanotubes with well-organized hexagonal nanowalls and sealed layouts: applications for lithium ion battery anodes. J Phys Chem C.

[12] Wang H, Pan Q, Cheng Y, Zhao J, Yin G (2009). Evaluation of ZnO nanorod arrays with dandelion-like morphology as negative electrodes for lithium-ion batteries. Electrochim Acta.

[13] Ahmad M, Yingying S, Nisar A, Sun H, Shen W, Wei M (2011). Synthesis of hierarchical flower-like ZnO nanostructures and their functionalization by Au nanoparticles for improved photocatalytic and high performance Li-ion battery anodes. J Mater Chem.

[14] Liu J, Li Y, Ding R, Jiang J, Hu Y, Ji X (2009). Carbon/ZnO nanorod array electrode with significantly improved lithium storage capability. J Phys Chem C.

[15] Shen X, Mu D, Chen S, Wu B, Wu F (2013). Enhanced electrochemical performance of ZnO-loaded/porous carbon composite as anode materials for lithium ion batteries. ACS Appl Mater Interfaces.

[16] Yang SJ, Nam S, Kim T, Im JH, Jung H, Kang JH (2013). Preparation and exceptional lithium anodic performance of porous carbon-coated ZnO quantum dots derived from a metal-organic framework. J Am Chem Soc.

[17] Abbas SM, Hussain ST, Ali S, Ahmad N, Ali N, Abbas S (2013). Structure and electrochemical performance of ZnO/CNT composite as anode material for lithium-ion batteries. J Mater Sci.

[18] Song WT, Xie J, Liu SY, Zheng YX, Cao GS, Zhu TJ (2012). Graphene decorated with ZnO nanocrystals with improved electrochemical properties prepared by a facile in situ hydrothermal route. Int J Electrochem Sci.

[19] Cai Z, Xu L, Yan M, Han C, He L, Hercule KM (2014). Manganese oxide/carbon yolk-shell nanorod anodes for high capacity lithium batteries. Nano Lett.

[20] Yan M, Wang F, Han C, Ma X, Xu X, An Q (2013). Nanowire templated semihollow bicontinuous graphene scrolls: designed construction, mechanism, and enhanced energy storage performance. J Am Chem Soc.

[21] Xu W, Zhao K, Niu C, Zhang L, Cai Z, Han C (2014). Heteorogeneous branched core-shell SnO_2_-PANI nanorod arrays with mechanical integrity and three dimensional electron transport for lithium batteries. Nano Energy.

[22] Chen Y, Ma J, Yu L, Li Q, Wang T (2012). Mesoporous SnO_2_ nanospheres formed via a water-evaporating process with superior eletrochemical properties. Cryst Eng Comm.

[23] Wang H, Wu Y, Bai Y, Zhou W, An Y, Li J (2011). The self-assembly of porous microspheres of tin dioxide octahedral nanoparticles for high performance lithium ion battery anode materials. J Mater Chem.

[24] Xu JS, Zhu YJ (2012). Monodisperse Fe_3_O_4_ and γ-Fe_2_O_3_ magnetic mesoporous microspheres as anode materials for lithium-ion batteries. ACS Appl Mater Interfaces.

[25] Liu N, Lu Z, Zhao J, McDowell MT, Lee HW, Zhao W (2014). A pomegranate-inspired nanoscale design for large-volume-change lithium battery anodes. Nat Nanotechnol.

[26] Kim C, Noh M, Choi M, Cho J, Park B (2005). Critical size of a nano SnO_2_ electrode for Li-secondary battery. Chem Mater.

[27] Waser O, Hess M, Güntner A, Novák P, Pratsinis SJ (2013). Size controlled CuO nanoparticles for Li-ion batteries. J Power Sources.

[28] He M, Yuan L, Hu X, Zhang W, Shu J, Huang YA (2013). SnO_2_@carbon nanocluster anode material with superior cyclability and rate capability for lithium-ion batteries. Nanoscale.

[29] Chen JS, Cheah YL, Chen YT, Jayaprakash N, Madhavi S, Yang YH (2009). SnO_2_ nanoparticles with controlled carbon nanocoating as high-capacity anode materials for lithium-ion batteries. J Phys Chem C.

[30] Hassoun J, Derrien G, Panero S, Scrosati B (2008). A nanostructured Sn-C composite lithium battery electrode with unique stability and high electrochemical performance. Adv Mater.

[31] Jayaprakash N, Jones WD, Moganty SS, Archer LA (2012). Composite lithium battery anodes based on carbon@Co_3_O_4_ nanostructures: synthesis and characterization. J Power Sources.

[32] Amine K, Yasuda H, Yamachi M (2000). Olivine LiCoPO_4_ as 4.8 V electrode material for lithium batteries. Electrochem Solid-State Lett.

[33] Jin YH, Min KM, Shim HW, Seo SD, Hwang IS, Park KS (2012). Facile synthesis of nano-Li_4_Ti_5_O_12_ for high-rate Li-ion battery anodes. Nanoscale Res Lett.

[34] Yoshio M, Wang H, Fukuda K, Umeno T, Dimov N, Ogumi Z (2002). Carbon-coated Si as a lithium-ion battery anode material. J Electrochem Soc.

[35] Nam S, Kim S, Wi S, Choi H, Byun S, Choi S (2012). The role of carbon incorporation in SnO_2_ nanoparticles for Li rechargeable batteries. J Power Sources.

[36] Moon T, Kim C, Hwang ST, Park B (2006). Electrochemical properties of disordered-carbon-coated SnO_2_ nanoparticles for Li rechargeable batteries. Electrochem Solid-State Lett.

[37] Yang L, Liu L, Zhu Y, Wang X, Wu Y (2012). Preparation of carbon coated MoO_2_ nanobelts and their high performance as anode materials for lithium ion batteries. J Mater Chem.

[38] Zhu J, Lei D, Zhang G, Li Q, Lu B, Wang T (2013). Carbon and graphene double protection strategy to improve the SnO_x_ electrode performance anodes for lithium-ion batteries. Nanoscale.

[39] Xu LL, Bain SW, Song KL (2014). Graphene sheets decorated with ZnO nanoparticles as anode materials for lithium ion batteries. J Mater Sci.

[40] Hsieh CT, Lin CY, Chen YF, Lin JS (2013). Synthesis of ZnO@graphene composites as anode materials for lithium ion batteries. Electrochim Acta.

[41] Yu M, Shao D, Lu F, Sun X, Sun H, Hu T (2013). ZnO/graphene nanocomposite fabricate by high energy ball milling with greatly enhanced lithium storage capability. Electrochem Commun.

[42] Zhou W, Zhu J, Cheng C, Liu J, Yang H, Cong C (2011). General strategy toward graphene@metal oxide core–shell nanostructures for high-performance lithium storage. Energy Environ Sci.

[43] Wi S, Kim J, Nam S, Kang J, Lee S, Woo H (2014). Enhanced rate capability of LiMn_0.9_ Mg_0.1_Po_4_ nanoplates by reduced graphene oxide/carbon double coating for Li-ion batteries. Curr Appl Phys.

[44] Park JS, Meng X, Elam JW, Hao S, Wolverton C, Kim C (2014). Ultrathin lithium-ion conducting coatings for increased interfacial stability in high voltage lithium-ion batteries. Chem Mater.

[45] Zhang Q, Chou TP, Russo B, Jenekhe SA, Cao G (2008). Aggregation of ZnO nanocrystallites for high conversion efficiency in dye-sensitized solar cells. Angew Chem.

[46] Kim C, Kim J, Choi H, Nahm C, Kang S, Lee S (2013). The effect of TiO_2_-coating layer on the performance in nanoporous ZnO-based dye-sensitized solar cells. J Power Sources.

[47] Hummers W, Offeman R (1958). Preparation of graphitic oxide. J Am Chem Soc.

[48] Li D, Müller MB, Gilje S, Kaner RB, Wallace GG (2008). Processable aqueous dispersions of graphene nanosheets. Nat Nanotechnol.

[49] Yang S, Feng X, Ivanovici S, Müllen K (2010). Fabrication of graphene-encapsulated oxide nanoparticles: towards high-performance anode materials for lithium storage. Angew Chem Int Ed.

[50] Chen D, Ji G, Ma Y, Lee JW, Lu J (2011). Graphene-encapsulated hollow Fe_3_O_4_ nanoparticle aggregates as a high-performance anode material for lithium ion batteries. ACS Appl Mater Interfaces.

[51] Chen JS, Wang Z, Dong XC, Chen P, Lou XW (2011). Graphene-wrapped TiO_2_ hollow structures with enhanced lithium storage capabilities. Nanoscale.

[52] Oh Y, Nam S, Wi S, Kang J, Hwang T, Lee S (2014). Effective wrapping of graphene on individual Li_4_Ti_5_O_12_ grains for high-rate Li-ion batteries. J Mater Chem A.

[53] Kim B, Lee JG, Choi M, Cho J, Park B (2004). Correlation between local strain and cycle-life performance of AlPO_4_-coated LiCoO_2_ cathodes. J Power Sources.

[54] Cho J, Kim TJ, Park B (2002). The effect of a metal-oxide coating on the cycling behavior at 55°C in orthorhombic LiMnO_2_ cathode materials. J Electrochem Soc.

[55] Yang GZ, Song HW, Cui H, Liu YC, Wang CX (2013). Ultrafast Li-ion battery anode with superlong life and excellent cycling stability from strongly coupled ZnO nanoparticle/conductive nanocarbon skeleton hybrid materials. Nano Energy.

[56] Tao HC, Fan LZ, Qu X (2012). Facile synthesis of ordered porous Si@C nanorods as anode materials for Li-ion batteries. Electrochim Acta.

[57] Yuan T, Yu X, Cai R, Zhou Y, Shao Z (2010). Synthesis of pristine and carbon-coated Li_4_Ti_5_O_12_ and their low-temperature electrochemical performance. J Power Sources.

[58] Qian J, Zhou M, Cao Y, Ai X, Yang H (2010). Template-free hydrothermal synthesis of nanoembossed mesoporous LiFePO_4_ microspheres for high-performance lithium-ion batteries. J Phys Chem C.

[59] Chan CK, Patel RN, O’Connell MJ, Korgel BA, Cui Y (2010). Solution-grown silicon nanowires for lithium-ion battery anodes. ACS Nano.

[60] Su Y, Li S, Wu D, Zhang F, Liang H, Gao P (2012). Two-dimensional carbon-coated graphene/metal oxide hybrids for enhanced lithium storage. ACS Nano.

[61] Zhou G, Wang DW, Li L, Li N, Li F, Cheng HM (2013). Nanosize SnO_2_ confined in the porous shells of carbon cages for kinetically efficient and long-term lithium storage. Nanoscale.

[62] Li C, Yu Z, Fang S, Wang H, Gui Y, Xu J (2009). Preparation and performance of ZnO nanoparticle aggregation with porous morphology. J Alloys Componds.

