# Supplementary material for: Reduced graphene oxide/carbon double-coated 3-D porous ZnO aggregates as high-performance Li-ion anode materials
Source: Nanoscale Res Lett. 2015 May 1;10:204. doi: 10.1186/s11671-015-0902-7 (PMC4422825; doi:10.1186/s11671-015-0902-7)
Supplement: Additional file 1: — Supporting Information. Table S1. BET surface area and average pore size of the C/ZnO, RGO/ZnO, and RGO/C/ZnO. Figure S1. SEM image of the bare ZnO aggregates. Figure S2. SEM images of the (a) RGO/ZnO aggregates and (b) RGO/C/ZnO aggregates. Figure S3. N2 adsorption/desorption isotherms of the C/ZnO, RGO/ZnO, and RGO/C/ZnO. The inset shows the pore-size distribution of these samples. Figure S4. Cyclic-voltammetry of (a) C/ZnO and (b) RGO/C/ZnO (0.001 to 3.0 V with the scan rate of 0.1 mV/s). [file 11671_2015_902_MOESM1_ESM.docx]

Supporting Information

**Reduced Graphene Oxide/Carbon Double-Coated**

**3-D Porous ZnO Aggregates as**

**High-Performance Li-Ion Anode Materials**

**Sungun Wi,^1,+^ Hyungsub Woo,^1,+^ Sangheon Lee,^1^ Joonhyeon Kang,^1^ Jaewon Kim,^1^**

**Subin An,^1^ Chohui Kim,^1^ Seunghoon Nam,^1^ Chunjoong Kim,^2^ and Byungwoo Park^1,*^**

^1^WCU Hybrid Materials Program, Department of Materials Science and Engineering,

Research Institute of Advanced Materials, Seoul National University, Seoul 151-744, Korea

^2^School of Materials Science and Engineering,

Chungnam National University, Daejeon 305-764, Korea

Table S1. BET surface area and average pore size of the C/ZnO, RGO/ZnO, and RGO/C/ZnO.

| Sample | BET surface area (m^2^/g) | Average pore size (nm) |
| --- | --- | --- |
| C/ZnO | 62.3 | 6.9 |
| RGO/ZnO | 32.0 | 15.3 |
| RGO/C/ZnO | 62.7 | 6.6 |

^*^ E-mail: [byungwoo@snu.ac.kr](mailto:byungwoo@snu.ac.kr); Phone: +82-2-880-8319; Fax: +82-2-885-9671.

^+^ Two authors contributed equally to this work.


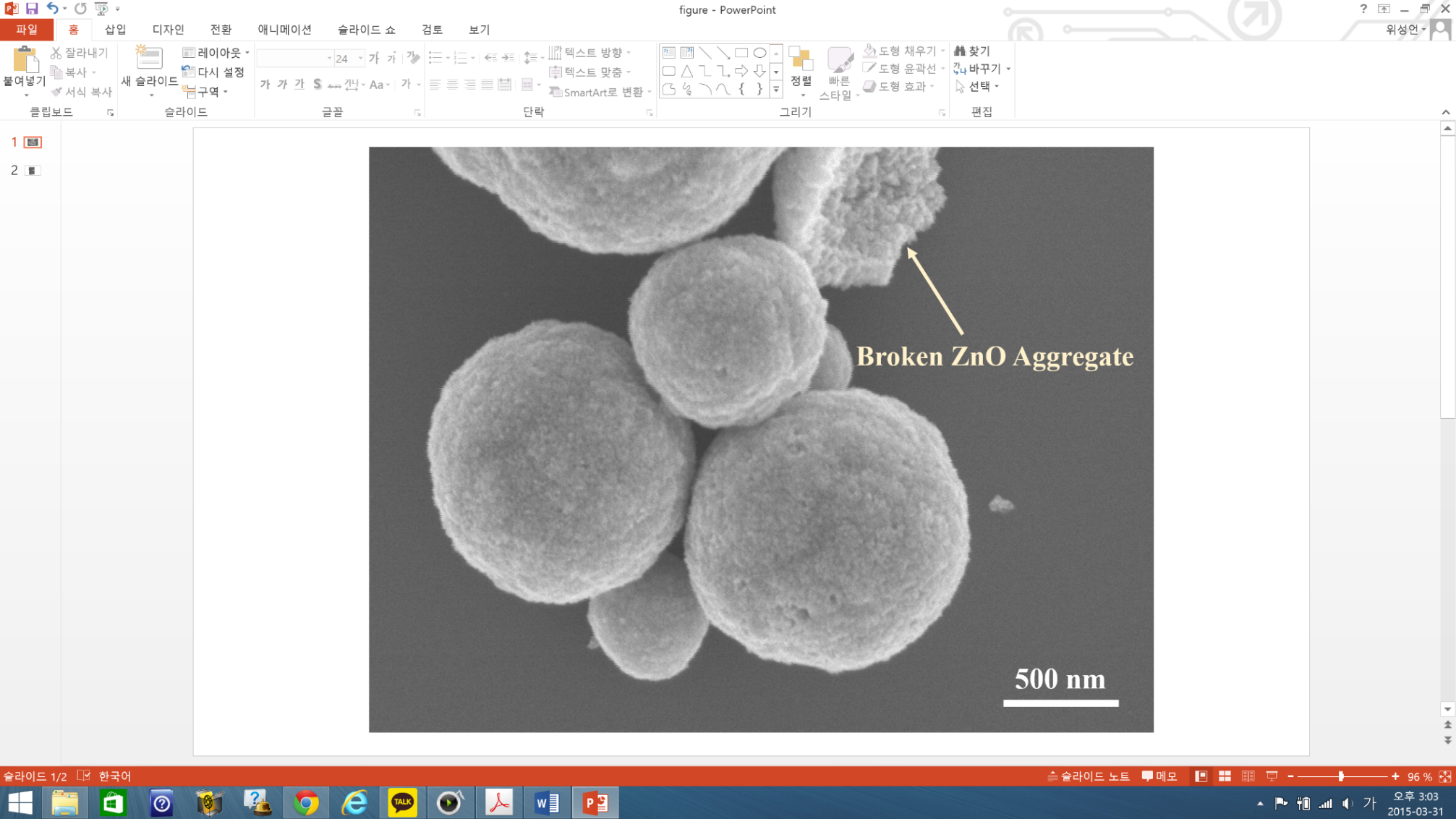


Fig. S1. SEM image of the bare ZnO aggregates.


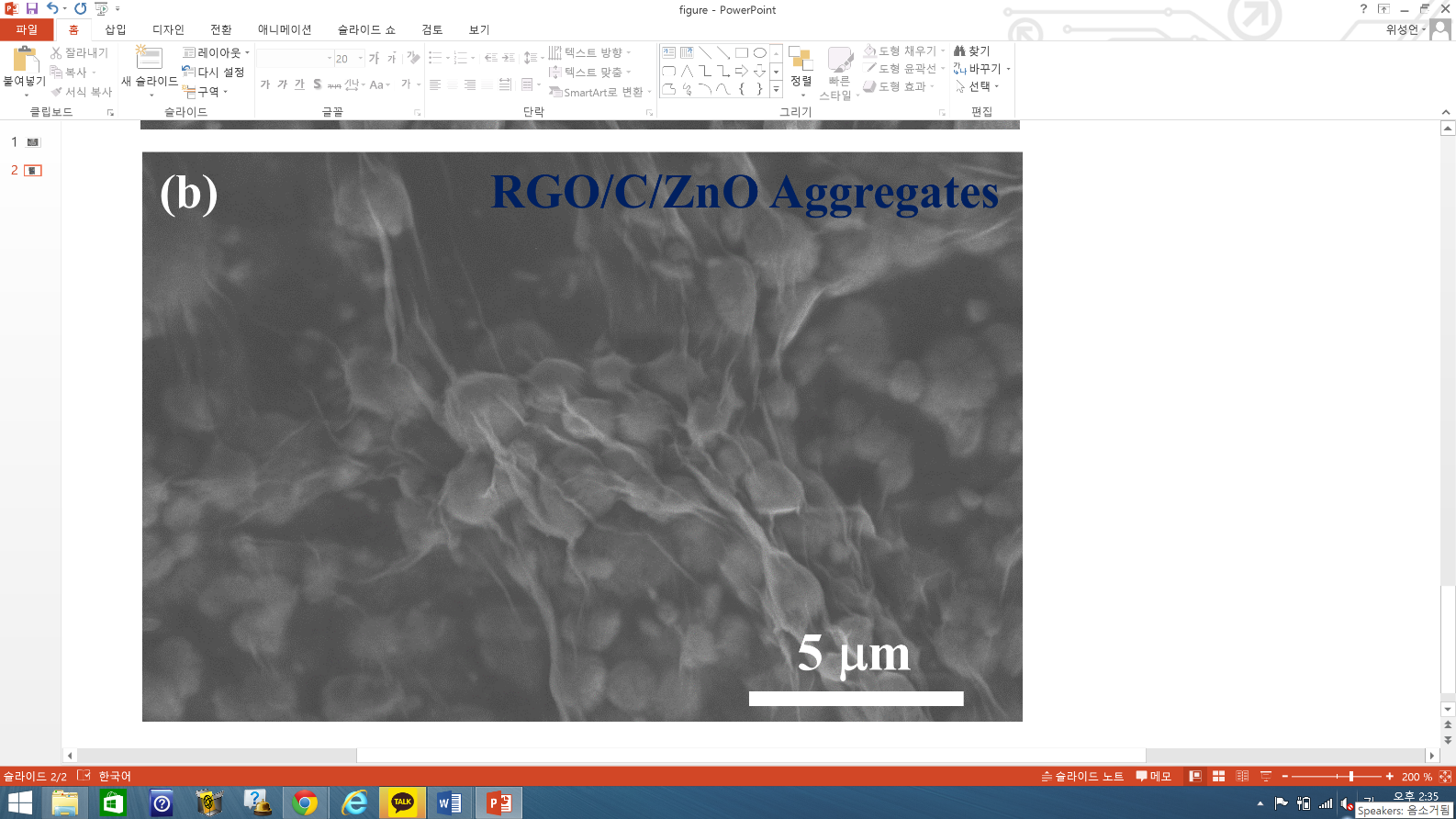

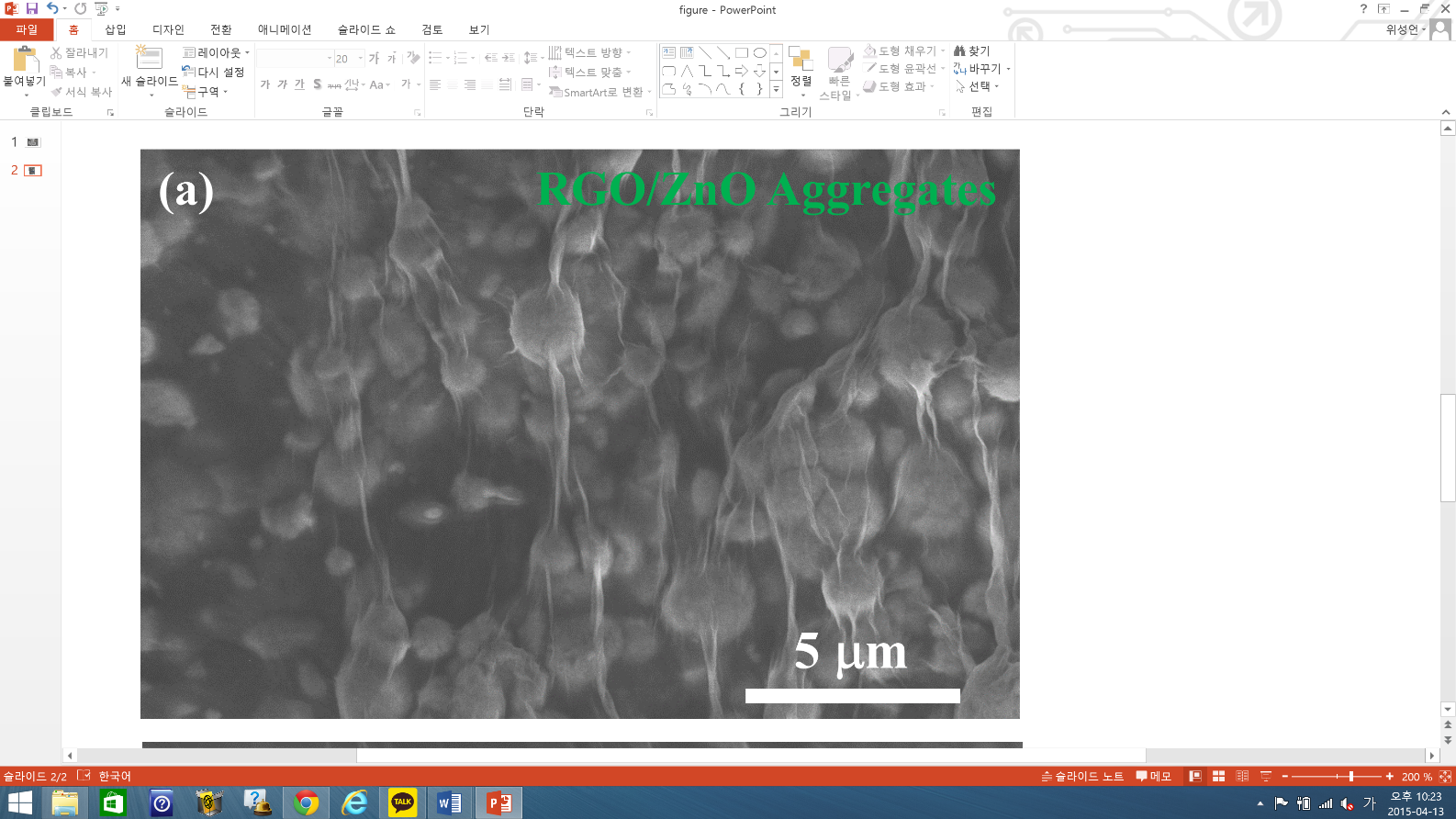


Fig. S2. SEM images of the (a) RGO/ZnO aggregates and (b) RGO/C/ZnO aggregates.

Fig. S3. N_2_ adsorption/desorption isotherms of the C/ZnO, RGO/ZnO, and RGO/C/ZnO. The inset shows the pore-size distribution of these samples.

Fig. S4. Cyclic-voltammetry of (a) C/ZnO and (b) RGO/C/ZnO (0.001 - 3.0 V with the scan rate of 0.1 mV/s).
